# Diversity of Methicillin-Resistant *Staphylococcus aureus* (MRSA) Strains Isolated from Inpatients of 30 Hospitals in Orange County, California

**DOI:** 10.1371/journal.pone.0062117

**Published:** 2013-04-24

**Authors:** Lyndsey O. Hudson, Courtney R. Murphy, Brian G. Spratt, Mark C. Enright, Kristen Elkins, Christopher Nguyen, Leah Terpstra, Adrijana Gombosev, Diane Kim, Paul Hannah, Lydia Mikhail, Richard Alexander, Douglas F. Moore, Susan S. Huang

**Affiliations:** 1 Department of Infectious Disease Epidemiology, Imperial College London, London, United Kingdom; 2 School of Social Ecology and Division of Infectious Diseases, University of California Irvine School of Medicine, Irvine, California, United States of America; 3 Division of Infectious Diseases and Health Policy Research Institute, University of California Irvine School of Medicine, Irvine, California, United States of America; 4 Orange County Health Care Agency, Santa Ana, California, United States of America; Rockefeller University, United States of America

## Abstract

There is a need for a regional assessment of the frequency and diversity of MRSA to determine major circulating clones and the extent to which community and healthcare MRSA reservoirs have mixed. We conducted a prospective cohort study of inpatients in Orange County, California, systematically collecting clinical MRSA isolates from 30 hospitals, to assess MRSA diversity and distribution. All isolates were characterized by *spa* typing, with selective PFGE and MLST to relate *spa* types with major MRSA clones. We collected 2,246 MRSA isolates from hospital inpatients. This translated to 91/10,000 inpatients with MRSA and an Orange County population estimate of MRSA inpatient clinical cultures of 86/100,000 people. *spa* type genetic diversity was heterogeneous between hospitals, and relatively high overall (72%). USA300 (t008/ST8), USA100 (t002/ST5) and a previously reported USA100 variant (t242/ST5) were the dominant clones across all Orange County hospitals, representing 83% of isolates. Fifteen hospitals isolated more t008 (USA300) isolates than t002/242 (USA100) isolates, and 12 hospitals isolated more t242 isolates than t002 isolates. The majority of isolates were imported into hospitals. Community-based infection control strategies may still be helpful in stemming the influx of traditionally community-associated strains, particularly USA300, into the healthcare setting.

## Introduction

Methicillin-resistant *Staphylococcus aureus* (MRSA) is a major global cause of morbidity and mortality, imposing serious economic costs on patients and hospitals [1]–[3]. Prior to the mid-1990s, MRSA was largely a healthcare-associated pathogen, causing infection predominantly in people with frequent or recent contact with healthcare facilities (healthcare-associated MRSA [HA-MRSA]). In the US, MRSA carriage (both asymptomatic and symptomatic) is estimated at 6–12% in general hospital patient populations and 9–24% in intensive care units (ICUs) [4]–[6]. HA-MRSA has long been the primary cause of MRSA infections, but community-associated MRSA (CA-MRSA), which often causes infections among healthy children and young adults with no exposure to the healthcare setting, has become increasingly prevalent across the globe, particularly in the US [7]–[10]. While well documented in the community, there is increasing evidence that CA-MRSA has become established in many healthcare MRSA settings [11]–[14]. CA-MRSA has caused outbreaks in the hospital setting since 2003, often in pediatrics and obstetrics where HA-MRSA prevalence is low and community influx of patients without prior healthcare exposure is common [13]. Furthermore, some reports suggest traditionally CA-MRSA may be replacing traditionally HA-MRSA in hospitals [15]–[18].

USA300 (t008/ST8) is the predominant, traditionally CA-MRSA clone in the US that has rapidly disseminated and replaced USA400 (ST1 and *spa* types t127, t128 and t1178) since its isolation in 2000. Traditional CA-MRSA such as USA300 have characteristics that may offer a selective advantage over HA-MRSA, making it a strong competitor in the healthcare setting. While the genetic backgrounds and epidemiology of MRSA are rapidly evolving, in general CA-MRSA contain a smaller SCC*mec* element (usually type IV) than HA-MRSA (usually SCC*mec* types I-III), and also harbor fewer antibiotic resistance genes than healthcare-associated strains, which may result in a fitness benefit, and have a higher growth rate *in vitro* that may lead to successful colonization by outcompeting HA-MRSA [19]. Furthermore, the linkage of the arginine catabolic mobile element with SCC*mec* type IV in USA300 likely also confers increased fitness and/or pathogenicity [20]. Finally, greater expression of regulatory genes associated with the virulence factors panton-valentine leukocidin (PVL) and alpha-toxin has been shown in USA300 versus USA400 isolates, which may contribute to the invasiveness of USA300 [21], although there is evidence from neonatal units that CA-MRSA do not need PVL to cause nosocomial infections [13], [22]–[24].

In addition, as CA-MRSA strains move into the healthcare setting and are exposed to nosocomial antibiotic pressure, they have developed greater antibiotic resistance [25], [26]. Even CA-MRSA causing skin infections are becoming more resistant, although such multiple resistance remains low in many US centers [25], [27]–[29]. In one US study, USA300 isolates classified as healthcare-associated were significantly more likely to be ciprofloxacin-resistant than USA300 isolates classified as community-associated [30], and another study reported a USA300 isolate with intermediate vancomycin susceptibility and reduced daptomycin susceptibility from a hospital in San Francisco in 2007 [31]. As traditionally CA-MRSA strains continue to encroach on healthcare MRSA reservoirs, they may come to resemble the antibiotic resistance profiles of traditionally HA-MRSA and produce healthcare-associated infections [13], [32]–[34]. While it is not clear if CA-MRSA cause more severe disease in the healthcare setting and whether they are more transmissible than HA-MRSA, their higher fitness and growth rate could lead to increasing prevalence in hospitals [35].

Most prior studies of CA-MRSA penetration into hospital reservoirs involve a single center, although nationally representative data have been presented recently [17], [18]. Regional evaluation of healthcare facilities may provide further information about the extent of reservoir mixing of traditional CA-MRSA and HA-MRSA strains across community and academic healthcare facilities, as well as pediatric hospitals and long-term acute care facilities. A prospective, population-based study of clinical MRSA isolates across nine medical centers in San Francisco, California, found that USA300 was the predominant clone in both the community and hospital setting [36]. While this city-based study collected almost 4000 MRSA isolates, only a fifth of these were selected for molecular analysis, with the primary goal to determine clonal groupings based upon the isolate collection date (hospital-onset or community-onset). Further comprehensive evaluations of the diversity of isolates within and across clonal complexes will provide valuable information about how strains are evolving and being shared across facilities. A better understanding of the frequency and diversity of community- and healthcare-associated MRSA clones may inform strategies to prevent MRSA transmission and disease in the US.

We therefore conducted a prospective cohort study of inpatients in a large metropolitan county, covering 30 medical facilities and strain typing all MRSA isolates collected from both adult and pediatric patients, to investigate the frequency and genetic diversity of MRSA at a population level.

## Materials and Methods

### Study

We conducted the study as described previously [37]. This study was approved by the Institutional Review Board of the University of California Regents, and a waiver of informed consent was granted.

### Isolate Collection

Clinical (non-screening) isolates of MRSA from unique patients were collected from hospital microbiology laboratories between October 2008 and April 2010. Hospitals were instructed to collect non-blood MRSA isolates from unique patients up to a total of 100 or for a duration of 12 months, whichever came first. In addition, hospitals were instructed to collect all blood isolates from unique patients for the same 12-month period as non-blood isolates. Isolates from patients not admitted to hospital were excluded from the study. Samples were processed as described previously [37].

### Specimen Data and Hospital Characteristics

Specimen data, hospital characteristics and the Orange County population estimate were obtained as described previously [37].

### Laboratory Methods and Molecular Typing

All laboratory methods and molecular typing (*spa* typing, multi locus sequence typing (MLST) and SmaI pulsed-field gel electrophoresis (PFGE)) were performed as described previously [37]. *spa* typing was performed on all collected isolates. MLST and PFGE were performed on a subset of isolates (N = 284), selected as described previously [37], to confirm strain types and assign isolates to the major US MRSA clones (USA100, USA300 etc).

### Definitions

Throughout this study the terms “traditionally CA-MRSA” and “traditionally HA-MRSA” were used to emphasize that, while certain clones were originally identified as community- or healthcare-associated, such clones may well be present and/or established in both settings today. We used this terminology in order to more clearly evaluate any mixing of MRSA reservoirs. We also classified MRSA isolates according to the timing of isolation with respect to patient admission date. Thus, community-onset MRSA was defined as MRSA isolated less than three days after admission, and hospital-onset MRSA as MRSA isolated three or more days after admission.

### Statistical Analyses

We calculated the number of hospitalized patients with MRSA clinical cultures among both the total population of Orange County and total annual admissions across all 30 hospitals, accounting for duration of isolate collection within each hospital. The number of community-onset and hospital-onset MRSA clinical cultures among the total Orange County population was also calculated. χ^2^ tests were performed to compare the number of isolates belonging to *spa* types t008 and t002/t242 between hospitals. One-sample z-tests for equality of proportions were conducted, to compare the number of isolates belonging to *spa* types t008 and t002/t242 within each hospital, as well as the number of t002 and t242 isolates within each hospital. Simpson’s index of diversity (1−*D*) was used to estimate inter- and intra-hospital genetic diversity of the MRSA strains collected, as well as the genetic diversity of the two major *spa*-CCs. 1−*D* gives an unbiased measure of the probability of drawing two different *spa* types given the distribution of *spa* types in a sample [38]. 95% confidence intervals (CIs) were calculated as described previously [39]. For comparison of diversity indices, a significant difference (*p*<0.05) was determined by non-overlapping 95% CIs. Pearson’s correlation coefficients were computed to determine the relationship between hospital-level and isolate variables, and genetic diversity. Due to the small sample size (28 hospitals; two were excluded as they collected <10 MRSA isolates and thus their diversity estimates were unreliable) and the number of potential predictor variables for genetic diversity, variables were considered for entry into a bootstrapped multiple linear regression model based on a combination of their correlation coefficient and current knowledge regarding their association with MRSA. Only variables with *p*<0.1 in correlation tests were considered for the exploratory model. All statistical tests were performed using STATA (release 11, StataCorp 2009).

## Results

### Overview

Between October 2008 and April 2010, 2,246 clinical MRSA isolates were collected from 30 Orange County hospitals. Annual population incidence of clinical inpatient MRSA isolates in Orange County was estimated at 86/100,000 people, with inpatient risk estimated at 91/10,000 admissions. Annual population incidence of clinical inpatient MRSA isolates in Orange County that were community-onset was estimated at 60/100,000 people (62/10,000 admissions); incidence of those that were hospital-onset was estimated at 25/100,000 people (26/10,000 admissions). Most clinical MRSA isolates were isolated from wounds or abscesses (47%), in non-intensive care units (non-ICUs; 84%), and were community-onset (72%). Median patient age was 64 (IQR, 44–79; 13 missing values). Tables 1 and 2 give a summary overview of the participating hospitals and isolate characteristics.

**Table 1:** Summary of the 30 participating hospitals in Orange County, CA.

Characteristic	Median (IQR^a^)
Annual admissions	7868 (2819–16157)
% Hispanic patients	19.2 (11.4–32.9)
% Medicaid-insured patients	15.1 (5.8–34.6)
N MRSA isolates per hospital per month	4.7 (2.5–11)
N *spa* types per hospital	14 (7–17)
N LTAC-facilities^b^ (No. isolates (%))	6 (132 (5.9))

a IQR = interquartile range.

b LTAC = long-term acute care.

**Table 2:** Summary of the 2,246 clinical MRSA isolates from hospital inpatients in Orange County, CA.

Characteristic^a^	Total isolates, N	t008 isolates, N (%)	t242 isolates, N (%)	t002 isolates, N (%)
Blood Specimens	213	81 (38)	47 (22.1)	30 (14.1)
Non-Blood Specimens	2,016	939 (46.6)	431 (21.4)	316 (15.7)
*Wound/Abscess*	1047	669 (63.9)	113 (10.8)	101 (9.7)
*Sputum*	596	159 (26.7)	186 (31.2)	146 (24.5)
*Urine*	189	33 (17.5)	87 (46)	38 (20.1)
*Other*	184	78 (42.4)	45 (24.5)	31 (16.9)
ICU^b^ collection	374	121 (32.4)	100 (26.7)	85 (22.7)
Hospital-onset	627	239 (38.1)	160 (25.5)	122 (19.5)

a 17 missing values for specimen source.

b ICU = intensive care unit.

### spa Typing and MLST

Among the 2,246 MRSA isolates collected, 134 *spa* types were identified, including one non-typeable (NT) isolate and 28 *spa* types (1.6% of all isolates) that did not match any known *spa* sequence. These novel *spa* sequences were automatically submitted to the Ridom SpaServer via the Ridom StaphType software and were assigned new *spa* types. The isolate with the NT *spa* type was re-tested to confirm the result was not due to a processing error, and the sequence quality was deemed excellent by the StaphType software. The NT *spa* type bore closest resemblance to t008, with a missing nucleotide in the ninth repeat, making the repeat 23-bp long. This is surprising since it would put the *spa* coding region out of frame although others have reported *spa* repeats with an unexpected length (J. Rothganger, Ridom GmbH, personal communication). The NT *spa* type was submitted to Ridom for their records. The three most common *spa* types were t008, t242 and t002, representing 83% of all isolates collected (Table 3). The remaining 131 *spa* types represented 1.5% or less of all isolates (Tables 3 and S1).

**Table 3:** 10 most frequent *spa* types and their MLST types among isolates from hospital inpatients in Orange County, CA^a^.

Rank	*spa* type	MLST	Freq	%	Cumulative %
1	t008	8	1034	46	46
2	t242	5	478	21.3	67.3
3	t002	5	347	15.4	82.8
4	t024	8	33	1.5	84.2
5	t037	8	25	1.1	85.4
6	t045	5	22	1.0	86.3
7	t088	105	21	0.9	87.3
8	t127	474^b^	18	0.8	88.1
9	t306	5	14	0.6	88.7
10	t1737	5	12	0.5	89.2
–	Other	–	242	10.8	100.0

a The total number of *spa* types was 134, including one non-typeable isolate. Simpson’s index of diversity (1−*D*) value was 72% (95% CI 70%–73%). MLST = multilocus sequence type.

b t127 isolates were also ST1 and ST1900, both SLVs of ST474.

**Figure 1:**
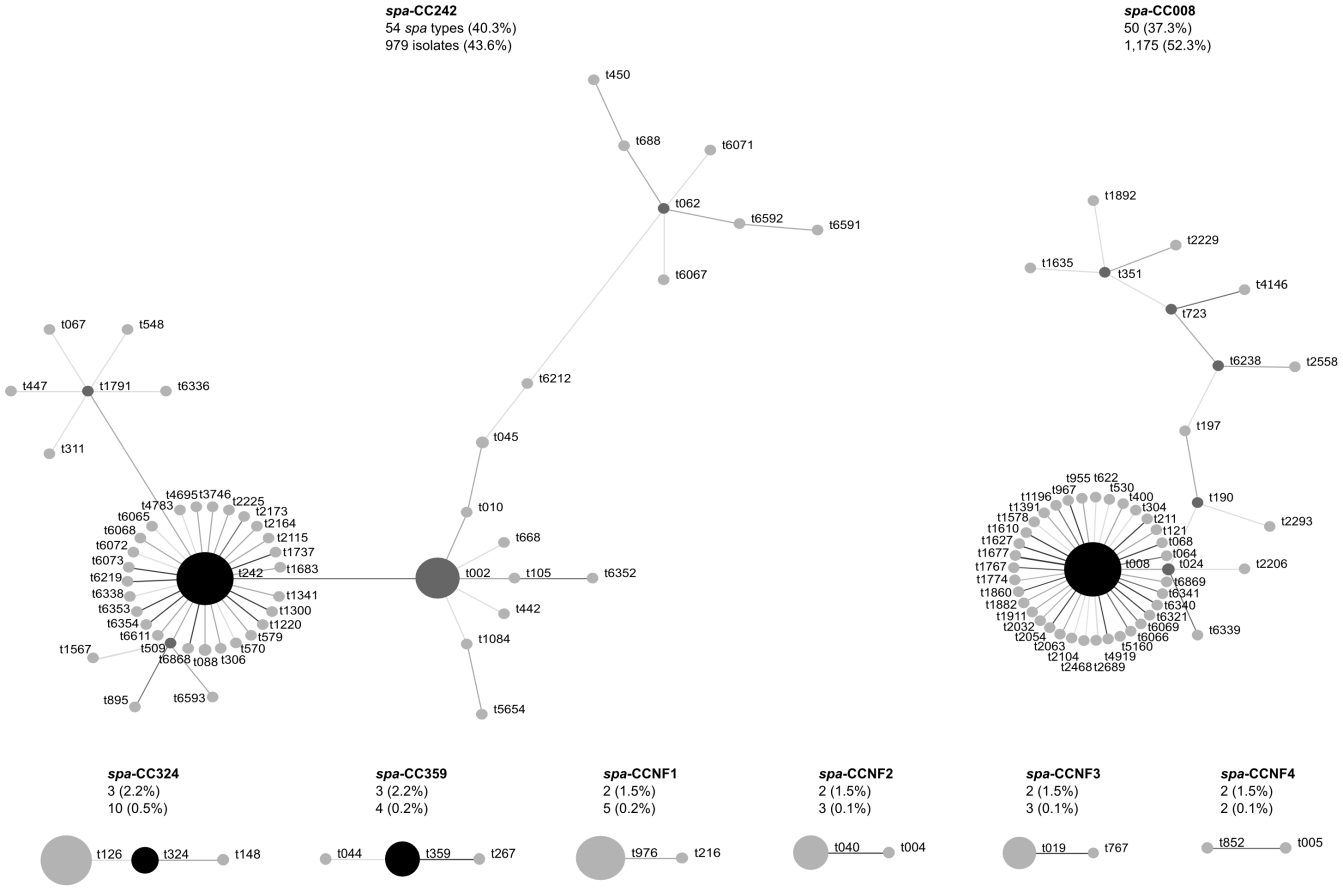
Relatedness of *spa* types among hospital MRSA isolates. Relatedness computed using the Based Upon Repeat Pattern (BURP) algorithm. Clusters of linked *spa* types correspond to *spa* clonal complexes (*spa*-CC). *spa* types are clustered into a *spa*-CC when their repeat patterns differ by no more than 4 repeats. BURP sums up ‘costs’ (a measure of relatedness based on the repeat pattern) to define a founder-score for each *spa* type in a *spa*-CC. The founder (black node) is the *spa* type with the highest founder-score in its *spa*-CC, and the subfounder (dark gray node) is the *spa* type with the second highest founder-score. *spa*-CC008 has founder t008, and *spa*-CCNF refers to a *spa*-CC with no founder. Each node represents a *spa* type. Node size represents the number of clustered strains that belong to that *spa* type. The shading of the branches represents the ‘costs’ (similarities in repeat patterns) between two *spa* types; the darker the branch, the lower the cost (more similar repeat patterns).

BURP analysis of the *spa* types clustered 96% of isolates into two large *spa*-CCs and 1.2% of isolates into six smaller *spa*-CCs (Figure 1). 78% of *spa* types were clustered into either *spa*-CC242 (founder t242) or *spa*-CC008 (founder t008), including 18 and 8 novel *spa* types, respectively. Under the BURP algorithm, *spa* types that differ from all other *spa* types in the sample by more than 4 repeats cannot reasonably be clustered into a *spa*-CC, and are termed singletons. Nine *spa* types (56 isolates) were classed as singletons, including two novel *spa* types. Six isolates represented six *spa* types that were less than five repeats in length and were excluded from BURP analysis because no reliable evolutionary history can be inferred from ‘short’ *spa* types [40]. The NT isolate could not be included in the BURP algorithm. Estimated genetic diversity of MRSA in Orange County hospitals using *spa* typing was high, at 72% (Table 3).

To confirm strain types, 284 isolates were selected for MLST. Among the 23 unique sequence types (STs) identified, ST5 (45%), ST8 (38%) and ST105 (4%) were the three most common, with the vast majority of isolates (90%) belonging to one of two major MLST CCs: CC5 (50%; four STs) and CC8 (40%; three STs) (Table 4). According to MLST, t008 isolates were ST8 and t002 isolates were ST5. t242 isolates were also identified as ST5 (Tables 3 and 4). PFGE of the subset of isolates (N = 284) confirmed that t008/ST8 isolates were the prototypic community clone USA300 and t002/ST5 and t242/ST5 isolates were predominantly the prototypic hospital clone USA100 (data not shown). *spa* type t242 differs from t002 by one *spa* repeat, as a result of a single nucleotide difference. The non-typeable *spa* isolate was ST8, with 64% of the novel *spa* types being ST5 and 36% ST8.

**Table 4:** Relatedness of MLST types of 284 hospital MRSA isolates according to eBURST^a^.

CC (no. of isolates)^b^	MLST	Associated *spa* types^c^
CC5 (142)	5	t242, t002, t045
	105	t088, t045
	225	t045
	840	t088
CC8 (114)	8	t008, t024, t037
	239	t037
	576	t1635
CC474 (9)	474	t127
	1900	t127
	1	t127
CCNF1 (4)	45	t004, t026, t040
	1811	t1081
CCNF2 (3)	59	t3424, t976
	87	t216
CCNF3 (2)	36	t018
	30	t019
Singletons (10)^d^	72	t126, t148, t324
	22	t005
	12	t160
	88	t5916
	97	t359
	188	t189
	635	t044

a MLST = multi-locus sequence typing; eBURST = Based Upon Related Sequence Types algorithm.

b CC = clonal complex. All members of a CC share identical alleles at six of the seven loci with at least one other member of the CC. CCNF refers to a CC with no predicted founder genotype.

c Only the three most common *spa* types are listed if more than three associated with that sequence type (ST).

d STs with allelic profiles that share less than six of their seven loci with all other STs in the dataset.

### Inter-hospital Differences

**Figure 2:**
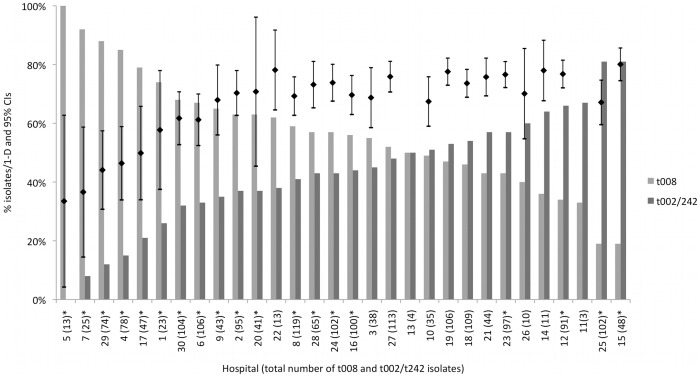
Relative proportion of isolates with *spa* type t008 versus *spa* types t002/242, by hospital. *indicates a significant difference at the 95% level in the relative proportion of isolates with *spa* type t008 and *spa* types t002/242 at that hospital. The black bars show the point estimates and 95% confidence intervals of hospital-specific genetic diversity expressed as Simpson’s index of diversity (1−*D*) of *spa* types (as a percentage). Diversity indices for hospitals 11 and 13 were excluded from the figure as these hospitals had *spa* type data for less than ten isolates (4 and 6 isolates in total, respectively. Diversity indices with non-overlapping 95% CIs were considered significantly different (*p*<0.05). The hospital-specific proportions of t008 among all *spa* types have been previously reported [59].

The estimated genetic diversity of MRSA within hospitals ranged from 33% to 79% (Figure 2). Percentage of blood specimens isolated per hospital and the median age of patients that specimens were collected from were positively correlated with genetic diversity within hospitals (r = 0.57, *p*<0.01 and r = 0.78, *p*<0.001 respectively). Significant negative correlations were found between genetic diversity of hospital MRSA isolates and the percentage of Medicaid-insured patients (r = −0.57, *p*<0.01), Hispanic patients (r = −0.38, *p* = 0.04) and wound/abscess specimens (r = −0.65, *p*<0.001) per hospital. Percentage of Hispanic patients and percentage of Medicaid-insured patients were highly correlated (r = 0.85, *p*<0.001), and since both are markers for patients from economically disadvantaged/high-density areas, the former was not considered for entry into the bootstrapped linear regression model. Only percentage of blood specimens and median patient age remained significantly correlated to genetic diversity in the exploratory regression model (coefficient = 0.82, bootstrap standard error = 0.24, normal-based 95% CI = 0.35–1.29, *p*<0.01 and coefficient = 0.44, bootstrap standard error = 0.08, normal-based 95% CI = 0.28–0.60, *p*<0.001 respectively).

The three most common *spa* types – t008, t242 and t002 - accounted for 65–95% of isolates at each hospital, showing that these *spa* types are consistently dominant across Orange County hospitals (Table S1). The proportion of t008 (USA300) isolates compared to t002/t242 (USA100) isolates varied significantly between hospitals (χ^2^ = 233.22, df = 29, *p*<0.001) (Figure 2). Four hospitals (13.3%) had significantly more t002/t242 (USA100) isolates, whereas 15 hospitals (50%) had significantly more t008 (USA300) isolates (*p*<0.05). Of the t002/t242 isolates, 12 (40%) hospitals had significantly more t242 isolates, whereas five hospitals (16.7%) had significantly more t002 isolates (*p*<0.05). One hospital isolated no t002/t242 isolates. The four hospitals with significantly more t002/t242 (USA100) isolates than t008 (USA300) isolates were medium to large non-teaching acute care hospitals. Diversity of *spa* types among *spa*-CC008 (1−*D = *22% (95% CI, 19–26%)) was significantly lower than diversity among *spa*-CC242 (1−*D* = 63% (95% CI, 61–66%)).

## Discussion

We conducted a prospective cohort study of inpatients in a large metropolitan county, collecting all clinical MRSA isolates from 30 of 31 hospitals in order to investigate the frequency and genetic diversity of MRSA at a population level, and provide further information about the extent of community and healthcare MRSA reservoir mixing. To our knowledge, this is the first study to assess MRSA isolates from a population-based sample across a large region. While Liu et al. conducted a large population-based study of clinical MRSA isolates in both hospital inpatients and outpatients, they sampled from a single city and characterized only 20% of all MRSA isolates collected [36]. Our countywide study was more comprehensive, encompassing 30 hospitals and characterizing all inpatient clinical MRSA isolates (over 2000).

Countywide, three *spa* types dominated clinical MRSA isolates. USA300 (t008/ST8), the traditionally community-associated clone prevalent in the US, was the most common clone, making up just under half of all clinical MRSA isolates. USA100 (t002/ST5), the traditionally healthcare-associated clone, was also common, but interestingly, t242/ST5 isolates were slightly more common than t002/ST5 isolates. Given the clinical similarities of t242 and t002 isolates found in our previous study [37], and that PFGE showed a sample of t242 and t002 isolates to be predominantly USA100, t242/ST5 likely represents a variant of USA100 that has become prevalent in Orange County hospitals [37]. t242 has been reported sporadically elsewhere, but was endemic in one hospital in Italy [41]–[44]. USA300 was the predominant clone in San Francisco hospitals in 2004–2005, followed by strains of the CC5 lineage [36].

Most *spa* types were closely related to either the USA300 or USA100 clone, creating two distinct *spa*-CCs. The remaining unrelated *spa* types were clustered into six small *spa*-CCs representing several traditionally community- and healthcare-associated clones, but occurred only sporadically. The largest of these *spa*-CCs represented ST72, an invasive community-associated clone that was reported in elderly patients in South Korea just before our isolate collection began [45]. According to the US 2010 Census (factfinder2.census.gov), 17.9% of the Orange County population is Asian of which approximately 2.9% are Korean. ST72 strains belong to CC8 and have also been reported in Australia and Europe [46]. The other *spa*-CCs represented clones including the traditionally community-associated ST59 (WA-MRSA 15, t976) and ST30 (USA1100/Southwest Pacific clone), plus the ST97 clone reported only once before in the US [46], [47]. MSSA from the CC97 lineage are often isolated from cattle but only occasionally from humans, and MRSA from this lineage are rare [46].

The remaining two small *spa*-CCs included the traditionally hospital-associated clones USA600 (ST45/Berlin clone) and EMRSA-15 (ST22), however the latter has recently been reported in the community setting [48]. Among the few isolates not belonging to a *spa*-CC was the traditionally HA-MRSA clone USA200 (ST36/EMRSA-16), isolates representing the pandemic ST239 clone and isolates representing strains of MLST CC1, a traditionally CA-MRSA lineage that includes USA400. Most isolates of this latter group were *spa* type t127 and ST474, a SLV of ST1. ST1/t127 is one of the most common CA-MRSA strains in the UK [49]. t127 was also recently reported among US isolates by Tenover et al [18]. While there is MRSA diversity in the Orange County population, USA300 and USA100 continue to dominate in hospitals, with most diversity caused by their close *spa*-type relatives.

Overall genetic diversity of MRSA in Orange County was relatively high, but heterogeneous between hospitals. This variation in diversity was mostly non-significant, with all hospitals dominated by the three most common *spa* types t008, t242 and t002. Diversity was significantly lower among *spa*-CC008 isolates than *spa*-CC242 isolates. Since the founder of *spa*-CC008 is the traditionally community-associated USA300, perhaps this clonal complex represents younger strains that have had less time to diversify compared to those in *spa*-CC242, which is largely represented by the traditionally healthcare-associated clone USA100. In an exploratory model, genetic diversity was significantly associated with older patient age and isolation of MRSA from blood specimens. Clones traditionally associated with the healthcare setting are typically isolated from older patients, whereas clones traditionally linked to the community setting, which have had less time to diversify, are associated with children and young adults [37]. Blood infections are commonly associated with the use of invasive medical devices such as indwelling catheters and are historically caused by healthcare-associated strains, which are more established and thus more diverse. This picture is rapidly changing however, with recent studies by O’Hara et al and Tenover et al demonstrating the increasing prevalence and dominance of USA300 isolates among invasive isolates [17], [18].

The vast majority of MRSA isolates were obtained within the first two days of hospitalization, suggesting that MRSA hospital reservoirs are mainly maintained by importation. Such community-onset isolates can include healthcare-associated carriage or infection isolates which are often found on readmission to hospitals [50], [51]. A history of healthcare exposure however does not exclude the possibility of MRSA acquisition and onset in the community [50].

The high penetration of USA300 into Orange County hospitals highlights the potential need for community-based strategies to be implemented in an effort to address the MRSA epidemic in the community and minimize the ability of traditional community-associated MRSA strains to become endemic in all hospitals. Whether all hospitals will eventually become dominated by traditional community-associated MRSA strains remains to be seen. t008 (USA300) is currently the most frequent strain in 80% of Orange County hospitals. The consequences of CA-MRSA continuing to infiltrate the healthcare setting include 1) the emergence of multidrug resistant CA-MRSA due to nosocomial antibiotic pressure [52], 2) the potential of increased virulence of healthcare-associated infections due to PVL-positive CA-MRSA strains, although studies suggest CA-MRSA are clinically similar to HA-MRSA once in the healthcare setting [34], and 3) the risk of hospital outbreaks due to the influx of CA-MRSA from the ever-expanding community reservoir [35], [52], [53].

A limitation of this study was that few individual level characteristics were available. Also, we could not account for variation among hospitals in obtaining clinical cultures. We excluded screening cultures since mandatory screening of high-risk inpatients was not in place in California until 2009 and capture would have been inconsistent across facilities. Therefore, our population estimate of MRSA isolates from hospital inpatients is likely an underestimate. In addition, our estimate should not be construed as a measure of MRSA infection among inpatients. Clinical isolates often represent carriage without infection. Due to the study design, we were unable to distinguish between infection and colonization isolates. Finally, our estimates of the indices of *spa* type diversity within hospitals may have been influenced by differing sample sizes [39].

PFGE is the gold standard for MRSA typing and is one of the most discriminatory typing methods for studying local MRSA epidemiology such as outbreaks and nosocomial transmission [54], [55]. Unfortunately, PFGE is laborious and time-consuming, making it unsuitable for high-throughput typing of large numbers of isolates. The sequence-based methods of MLST and *spa* typing were used in this study because they are simpler and quicker to perform and unambiguous. Because of the need to sequence seven loci, MLST is less efficient than *spa* typing for a large number of isolates as in this study. Consequently, MLST was only performed on a subset of the isolates. This was also true for PFGE. *spa* typing is a single-locus method, and its discriminatory power lies between that of PFGE and MLST, making it suitable for studying hospital outbreaks and transmission as well as clonal evolution [56], [57]. These reasons made *spa* typing the method of choice for chracterising the 2,246 MRSA isolates in this study. A recent study found that *spa* type t008 has high specifity and sensitivity with regards to identifying USA300 isolates (95 and 93% respectively) [58]. Furthermore, *spa* type t008 in combination with MLST ST8 was shown to be highly specific (98%), meaning that this combination of genetic markers rarely identified a non-USA300 isolate [58]. Reducing the cost of whole genome sequencing will in future provide the ultimate tool for the molecular epidemiology of MRSA [58].

This study was designed to assess the extent of MRSA reservoir mixing and to examine how this varies across hospitals within a single, large US county. When combining data from all hospitals, we found that t008 (USA300) and t002/t242 (USA100) isolates dominated the hospital setting, with t008 (USA300) as the single most prevalent clone. Nevertheless, when evaluating individual hospitals, we found that genetic diversity was high and substantial variation existed. Genetic diversity of MRSA appeared to be driven by *spa* types closely related to those of t002 and t242 (USA100). The USA100 variant t242/ST5 was more prevalent than t002/ST5 and found in most Orange County hospitals.

USA300 was originally recognized as a community-associated MRSA strain but is now well established alongside traditional healthcare-associated MRSA strains in many hospitals, including those in Orange County, making the distinction between community-associated and hospital-associated strains increasingly untenable. Nevertheless, in 20% of hospitals, USA300 was not yet the dominant strain, suggesting that community-based MRSA control strategies may still be helpful in stemming the influx of traditionally community-associated strains into healthcare settings.

## Supporting Information

Table S1 ***spa*** **type frequencies by hospital for the 2,246 clinical MRSA isolates collected from 30 hospitals in Orange County, CA.**
(DOCX)
