# Supplementary material for: Diversity of Methicillin-Resistant Staphylococcus aureus (MRSA) Strains Isolated from Inpatients of 30 Hospitals in Orange County, California
Source: PLoS One. 2013 Apr 24;8(4):e62117. doi: 10.1371/journal.pone.0062117 (PMC3634754; doi:10.1371/journal.pone.0062117)
Supplement: Table S1 — spa type frequencies by hospital for the 2,246 clinical MRSA isolates collected from 30 hospitals in Orange County, CA. (DOCX) [file pone.0062117.s001.docx]

Table S1. *spa* type frequencies by hospital for the 2,246 clinical MRSA isolates collected from 30 hospitals in Orange County, CA.

| **Hospital** | ***spa* type** | **Frequency** | **%** |
| --- | --- | --- | --- |
| 1 | t008 | 17 | 63.0 |
|  | t242 | 4 | 14.8 |
|  | t002 | 2 | 7.4 |
|  | t024 | 1 | 3.7 |
|  | t306 | 1 | 3.7 |
|  | t622 | 1 | 3.7 |
|  | t955 | 1 | 3.7 |
|  | Total | 27 | 100.0 |
| 2 | t008 | 60 | 50.4 |
|  | t242 | 21 | 17.6 |
|  | t002 | 14 | 11.8 |
|  | t024 | 4 | 3.4 |
|  | t045 | 3 | 2.5 |
|  | t088 | 3 | 2.5 |
|  | t127 | 3 | 2.5 |
|  | t306 | 2 | 1.7 |
|  | t324 | 2 | 1.7 |
|  | t010 | 1 | 0.8 |
|  | t1774 | 1 | 0.8 |
|  | t1911 | 1 | 0.8 |
|  | t211 | 1 | 0.8 |
|  | t6069 | 1 | 0.8 |
|  | t6071 | 1 | 0.8 |
|  | t688 | 1 | 0.8 |
|  | Total | 119 | 100.0 |
| 3 | t008 | 21 | 47.7 |
|  | t002 | 12 | 27.3 |
|  | t242 | 5 | 11.4 |
|  | t024 | 2 | 4.5 |
|  | t045 | 1 | 2.3 |
|  | t1882 | 1 | 2.3 |
|  | t6072 | 1 | 2.3 |
|  | t723 | 1 | 2.3 |
|  | Total | 44 | 100.0 |
| 4 | t008 | 66 | 72.5 |
|  | t242 | 11 | 12.1 |
|  | t024 | 2 | 2.2 |
|  | t5654 | 2 | 2.2 |
|  | t002 | 1 | 1.1 |
|  | t005 | 1 | 1.1 |
|  | t068 | 1 | 1.1 |
|  | t088 | 1 | 1.1 |
|  | t105 | 1 | 1.1 |
|  | t1220 | 1 | 1.1 |
|  | t1578 | 1 | 1.1 |
|  | t216 | 1 | 1.1 |
|  | t2689 | 1 | 1.1 |
|  | t668 | 1 | 1.1 |
|  | Total | 91 | 100.0 |
| 5 | t008 | 13 | 81.3 |
|  | t024 | 1 | 6.3 |
|  | t068 | 1 | 6.3 |
|  | t088 | 1 | 6.3 |
|  | Total | 16 | 100.0 |
| 6 | t008 | 71 | 59.2 |
|  | t002 | 19 | 15.8 |
|  | t242 | 16 | 13.3 |
|  | t088 | 3 | 2.5 |
|  | t2468 | 2 | 1.7 |
|  | t723 | 2 | 1.7 |
|  | t044 | 1 | 0.8 |
|  | t121 | 1 | 0.8 |
|  | t2104 | 1 | 0.8 |
|  | t211 | 1 | 0.8 |
|  | t324 | 1 | 0.8 |
|  | t622 | 1 | 0.8 |
|  | t852 | 1 | 0.8 |
|  | Total | 120 | 100.0 |
| 7 | t008 | 23 | 79.3 |
|  | t002 | 2 | 6.9 |
|  | t211 | 2 | 6.9 |
|  | t121 | 1 | 3.4 |
|  | t1610 | 1 | 3.4 |
|  | Total | 29 | 100.0 |
| 8 | t008 | 70 | 50.0 |
|  | t242 | 27 | 19.3 |
|  | t002 | 22 | 15.7 |
|  | t024 | 3 | 2.1 |
|  | t045 | 3 | 2.1 |
|  | t088 | 2 | 1.4 |
|  | t127 | 2 | 1.4 |
|  | t1737 | 2 | 1.4 |
|  | t026 | 1 | 0.7 |
|  | t037 | 1 | 0.7 |
|  | t068 | 1 | 0.7 |
|  | t126 | 1 | 0.7 |
|  | t1300 | 1 | 0.7 |
|  | t4783 | 1 | 0.7 |
|  | t548 | 1 | 0.7 |
|  | t6321 | 1 | 0.7 |
|  | t6352 | 1 | 0.7 |
|  | Total | 140 | 100.0 |
| 9 | t008 | 28 | 52.8 |
|  | t002 | 9 | 17.0 |
|  | t242 | 6 | 11.3 |
|  | t088 | 2 | 3.8 |
|  | t127 | 2 | 3.8 |
|  | t306 | 2 | 3.8 |
|  | t359 | 2 | 3.8 |
|  | t105 | 1 | 1.9 |
|  | t723 | 1 | 1.9 |
|  | Total | 53 | 100.0 |
| 10 | t008 | 17 | 45.9 |
|  | t242 | 10 | 27.0 |
|  | t002 | 8 | 21.6 |
|  | t126 | 1 | 2.7 |
|  | t127 | 1 | 2.7 |
|  | Total | 37 | 100.0 |
| 11 | t242 | 2 | 50.0 |
|  | t008 | 1 | 25.0 |
|  | t2689 | 1 | 25.0 |
|  | Total | 4 | 100.0 |
| 12 | t242 | 40 | 35.7 |
|  | t008 | 31 | 27.7 |
|  | t002 | 20 | 17.9 |
|  | t1774 | 3 | 2.7 |
|  | t045 | 2 | 1.8 |
|  | t088 | 2 | 1.8 |
|  | t1737 | 2 | 1.8 |
|  | t010 | 1 | 0.9 |
|  | t064 | 1 | 0.9 |
|  | t068 | 1 | 0.9 |
|  | t1196 | 1 | 0.9 |
|  | t121 | 1 | 0.9 |
|  | t1683 | 1 | 0.9 |
|  | t2032 | 1 | 0.9 |
|  | t311 | 1 | 0.9 |
|  | t579 | 1 | 0.9 |
|  | t6068 | 1 | 0.9 |
|  | t622 | 1 | 0.9 |
|  | t6591 | 1 | 0.9 |
|  | Total | 112 | 100.0 |
| 13 | t008 | 2 | 33.3 |
|  | t002 | 1 | 16.7 |
|  | t024 | 1 | 16.7 |
|  | t242 | 1 | 16.7 |
|  | t570 | 1 | 16.7 |
|  | Total | 6 | 100.0 |
| 14 | t002 | 5 | 33.3 |
|  | t008 | 4 | 26.7 |
|  | t088 | 2 | 13.3 |
|  | t242 | 2 | 13.3 |
|  | t024 | 1 | 6.7 |
|  | t1737 | 1 | 6.7 |
|  | Total | 15 | 100.0 |
| 15 | t002 | 20 | 30.3 |
|  | t242 | 19 | 28.8 |
|  | t008 | 9 | 13.6 |
|  | t037 | 6 | 9.1 |
|  | t018 | 3 | 4.5 |
|  | t010 | 1 | 1.5 |
|  | t067 | 1 | 1.5 |
|  | t088 | 1 | 1.5 |
|  | t1737 | 1 | 1.5 |
|  | t304 | 1 | 1.5 |
|  | t530 | 1 | 1.5 |
|  | t548 | 1 | 1.5 |
|  | t6066 | 1 | 1.5 |
|  | t6868 | 1 | 1.5 |
|  | Total | 66 | 100.0 |
| 16 | t008 | 56 | 48.3 |
|  | t242 | 27 | 23.3 |
|  | t002 | 17 | 14.7 |
|  | t045 | 2 | 1.7 |
|  | t127 | 2 | 1.7 |
|  | t018 | 1 | 0.9 |
|  | t024 | 1 | 0.9 |
|  | t037 | 1 | 0.9 |
|  | t064 | 1 | 0.9 |
|  | t121 | 1 | 0.9 |
|  | t2054 | 1 | 0.9 |
|  | t2558 | 1 | 0.9 |
|  | t5160 | 1 | 0.9 |
|  | t6070 | 1 | 0.9 |
|  | t622 | 1 | 0.9 |
|  | t6352 | 1 | 0.9 |
|  | t842 | 1 | 0.9 |
|  | Total | 116 | 100.0 |
| 17 | t008 | 37 | 69.8 |
|  | t002 | 5 | 9.4 |
|  | t242 | 5 | 9.4 |
|  | t1081 | 2 | 3.8 |
|  | t024 | 1 | 1.9 |
|  | t190 | 1 | 1.9 |
|  | t622 | 1 | 1.9 |
|  | t688 | 1 | 1.9 |
|  | Total | 53 | 100.0 |
| 18 | t008 | 50 | 38.8 |
|  | t242 | 40 | 31.0 |
|  | t002 | 19 | 14.7 |
|  | t127 | 4 | 3.1 |
|  | t045 | 3 | 2.3 |
|  | t018 | 1 | 0.8 |
|  | t024 | 1 | 0.8 |
|  | t068 | 1 | 0.8 |
|  | t121 | 1 | 0.8 |
|  | t160 | 1 | 0.8 |
|  | t1791 | 1 | 0.8 |
|  | t2229 | 1 | 0.8 |
|  | t306 | 1 | 0.8 |
|  | t442 | 1 | 0.8 |
|  | t548 | 1 | 0.8 |
|  | t6066 | 1 | 0.8 |
|  | t6073 | 1 | 0.8 |
|  | t688 | 1 | 0.8 |
|  | Total | 129 | 100.0 |
| 19 | t008 | 50 | 37.0 |
|  | t242 | 34 | 25.2 |
|  | t002 | 22 | 16.3 |
|  | t064 | 5 | 3.7 |
|  | t976 | 3 | 2.2 |
|  | t088 | 2 | 1.5 |
|  | t127 | 2 | 1.5 |
|  | t6065 | 2 | 1.5 |
|  | t6340 | 2 | 1.5 |
|  | t010 | 1 | 0.7 |
|  | t024 | 1 | 0.7 |
|  | t045 | 1 | 0.7 |
|  | t1220 | 1 | 0.7 |
|  | t126 | 1 | 0.7 |
|  | t1300 | 1 | 0.7 |
|  | t2302 | 1 | 0.7 |
|  | t4695 | 1 | 0.7 |
|  | t4919 | 1 | 0.7 |
|  | t509 | 1 | 0.7 |
|  | t586 | 1 | 0.7 |
|  | t622 | 1 | 0.7 |
|  | t723 | 1 | 0.7 |
|  | Total | 135 | 100.0 |
| 20 | t008 | 26 | 50.0 |
|  | t242 | 9 | 17.3 |
|  | t002 | 6 | 11.5 |
|  | t024 | 2 | 3.8 |
|  | t306 | 2 | 3.8 |
|  | t018 | 1 | 1.9 |
|  | t037 | 1 | 1.9 |
|  | t062 | 1 | 1.9 |
|  | t267 | 1 | 1.9 |
|  | t3424 | 1 | 1.9 |
|  | t570 | 1 | 1.9 |
|  | t6337 | 1 | 1.9 |
|  | Total | 52 | 100.0 |
| 21 | t008 | 19 | 35.8 |
|  | t242 | 15 | 28.3 |
|  | t002 | 10 | 18.9 |
|  | t304 | 2 | 3.8 |
|  | t306 | 2 | 3.8 |
|  | t018 | 1 | 1.9 |
|  | t045 | 1 | 1.9 |
|  | t121 | 1 | 1.9 |
|  | t1578 | 1 | 1.9 |
|  | t6212 | 1 | 1.9 |
|  | Total | 53 | 100.0 |
| 22 | t008 | 8 | 40.0 |
|  | t242 | 4 | 20.0 |
|  | t2468 | 2 | 10.0 |
|  | t002 | 1 | 5.0 |
|  | t024 | 1 | 5.0 |
|  | t037 | 1 | 5.0 |
|  | t6238 | 1 | 5.0 |
|  | t967 | 1 | 5.0 |
|  | t976 | 1 | 5.0 |
|  | Total | 20 | 100.0 |
| 23 | t008 | 42 | 35.0 |
|  | t242 | 35 | 29.2 |
|  | t002 | 20 | 16.7 |
|  | t037 | 7 | 5.8 |
|  | t1860 | 3 | 2.5 |
|  | t024 | 2 | 1.7 |
|  | t105 | 2 | 1.7 |
|  | t126 | 1 | 0.8 |
|  | t1627 | 1 | 0.8 |
|  | t1737 | 1 | 0.8 |
|  | t2173 | 1 | 0.8 |
|  | t6072 | 1 | 0.8 |
|  | t6338 | 1 | 0.8 |
|  | t6339 | 1 | 0.8 |
|  | t6354 | 1 | 0.8 |
|  | t895 | 1 | 0.8 |
|  | Total | 120 | 100.0 |
| 24 | t008 | 58 | 45.3 |
|  | t242 | 24 | 18.8 |
|  | t002 | 20 | 15.6 |
|  | t1737 | 4 | 3.1 |
|  | t037 | 3 | 2.3 |
|  | t062 | 3 | 2.3 |
|  | t1341 | 2 | 1.6 |
|  | t306 | 2 | 1.6 |
|  | t6353 | 2 | 1.6 |
|  | t024 | 1 | 0.8 |
|  | t1084 | 1 | 0.8 |
|  | t121 | 1 | 0.8 |
|  | t127 | 1 | 0.8 |
|  | t197 | 1 | 0.8 |
|  | t2225 | 1 | 0.8 |
|  | t4146 | 1 | 0.8 |
|  | t6067 | 1 | 0.8 |
|  | t6219 | 1 | 0.8 |
|  | t6592 | 1 | 0.8 |
|  | Total | 128 | 100.0 |
| 25 | t242 | 62 | 52.5 |
|  | t002 | 21 | 17.8 |
|  | t008 | 19 | 16.1 |
|  | t024 | 2 | 1.7 |
|  | t045 | 2 | 1.7 |
|  | t1341 | 2 | 1.7 |
|  | t1683 | 2 | 1.7 |
|  | t040 | 1 | 0.8 |
|  | t1737 | 1 | 0.8 |
|  | t1791 | 1 | 0.8 |
|  | t189 | 1 | 0.8 |
|  | t2032 | 1 | 0.8 |
|  | t3746 | 1 | 0.8 |
|  | t509 | 1 | 0.8 |
|  | t6593 | 1 | 0.8 |
|  | Total | 118 | 100.0 |
| 26 | t002 | 5 | 41.7 |
|  | t008 | 4 | 33.3 |
|  | t019 | 1 | 8.3 |
|  | t045 | 1 | 8.3 |
|  | t242 | 1 | 8.3 |
|  | Total | 12 | 100.0 |
| 27 | t008 | 59 | 41.3 |
|  | t002 | 33 | 23.1 |
|  | t242 | 21 | 14.7 |
|  | t105 | 4 | 2.8 |
|  | t024 | 2 | 1.4 |
|  | t037 | 2 | 1.4 |
|  | t045 | 2 | 1.4 |
|  | t067 | 2 | 1.4 |
|  | t126 | 2 | 1.4 |
|  | t6068 | 2 | 1.4 |
|  | t004 | 1 | 0.7 |
|  | t018 | 1 | 0.7 |
|  | t064 | 1 | 0.7 |
|  | t088 | 1 | 0.7 |
|  | t121 | 1 | 0.7 |
|  | t148 | 1 | 0.7 |
|  | t211 | 1 | 0.7 |
|  | t2164 | 1 | 0.7 |
|  | t2293 | 1 | 0.7 |
|  | t306 | 1 | 0.7 |
|  | t351 | 1 | 0.7 |
|  | t400 | 1 | 0.7 |
|  | t6611 | 1 | 0.7 |
|  | t895 | 1 | 0.7 |
|  | Total | 143 | 100.0 |
| 28 | t008 | 37 | 45.7 |
|  | t242 | 17 | 21.0 |
|  | t002 | 11 | 13.6 |
|  | t037 | 3 | 3.7 |
|  | t024 | 2 | 2.5 |
|  | NT | 1 | 1.2 |
|  | t040 | 1 | 1.2 |
|  | t045 | 1 | 1.2 |
|  | t064 | 1 | 1.2 |
|  | t1578 | 1 | 1.2 |
|  | t1911 | 1 | 1.2 |
|  | t2063 | 1 | 1.2 |
|  | t306 | 1 | 1.2 |
|  | t447 | 1 | 1.2 |
|  | t4919 | 1 | 1.2 |
|  | t767 | 1 | 1.2 |
|  | Total | 81 | 100.0 |
| 29 | t008 | 65 | 74.7 |
|  | t002 | 5 | 5.7 |
|  | t242 | 4 | 4.6 |
|  | t1635 | 2 | 2.3 |
|  | t211 | 2 | 2.3 |
|  | t024 | 1 | 1.1 |
|  | t127 | 1 | 1.1 |
|  | t1767 | 1 | 1.1 |
|  | t1892 | 1 | 1.1 |
|  | t2104 | 1 | 1.1 |
|  | t2115 | 1 | 1.1 |
|  | t2206 | 1 | 1.1 |
|  | t5916 | 1 | 1.1 |
|  | t6869 | 1 | 1.1 |
|  | Total | 87 | 100.0 |
| 30 | t008 | 71 | 59.2 |
|  | t002 | 17 | 14.2 |
|  | t242 | 16 | 13.3 |
|  | t622 | 3 | 2.5 |
|  | t019 | 1 | 0.8 |
|  | t024 | 1 | 0.8 |
|  | t027 | 1 | 0.8 |
|  | t088 | 1 | 0.8 |
|  | t121 | 1 | 0.8 |
|  | t1391 | 1 | 0.8 |
|  | t1567 | 1 | 0.8 |
|  | t1677 | 1 | 0.8 |
|  | t197 | 1 | 0.8 |
|  | t304 | 1 | 0.8 |
|  | t450 | 1 | 0.8 |
|  | t6336 | 1 | 0.8 |
|  | t6341 | 1 | 0.8 |
|  | Total | 120 | 100.0 |
